# The Influence of Actual Appraisals of Peers on the Self-Appraisals of Personality Traits for Chinese Late Adolescents: The Mediating Effect of Reflected Appraisals

**DOI:** 10.3389/fpsyg.2021.687482

**Published:** 2021-08-26

**Authors:** Caizhen Yue, Yihong Long, Zhiwen Yang, Qianguo Xiao, Weigang Pan

**Affiliations:** ^1^College of National Culture and Cognitive Science, Guizhou Minzu University, Guiyang, China; ^2^Laboratory of Emotion and Mental Health, Chongqing University of Arts and Sciences, Chongqing, China

**Keywords:** reflected appraisals, self-appraisals, others' actual appraisals, Big Five personality, mediating effect

## Abstract

Reflected appraisals refer to the perceptions of individuals of how they are perceived by others. Numerous studies in cultural psychology have revealed that individuals in the Eastern collectivist culture show an interdependent self-construal, which depends much on the social culture. Hence, the research on reflected appraisals in the Eastern culture can improve the understanding of how the social environment shapes the self-perception of an individual. In this study, we aimed to explore the relationships among self-appraisals, reflected appraisals, and actual appraisals of peers of the Big Five personality for Chinese late adolescents. Participants were divided into 16 groups, with two to four people of each group who were familiar with each other. Each participant was told to fill out the questionnaires of reflected appraisals, actual appraisals of peers, and self-appraisals. Through analyzing 164 sets of data, the results showed the following: (a) The scores of reflected appraisals are significantly lower than that of the actual appraisals of peers. (b) The relationships among the reflected appraisals, actual appraisals of peers, and self-appraisals are distinct on different personalities. For extroversion, there are significant medium- to high-degree relationships among the three types of appraisals; while for the agreeableness, conscientiousness, emotional stability, and openness, self-appraisals are highly correlated with reflected appraisals, and reflected appraisals show a low-degree correlation with the actual appraisals of peers. (c) Reflected appraisals play a mediating role between actual appraisals of peers and self-appraisals. Our study suggests that individuals in Chinese culture generally underestimate how their peers perceive them. Furthermore, actual appraisals of peers affect the self-concepts of individuals through reflected appraisals. This study revealed the unique personality feature of self-modesty under the background of Chinese culture and the importance of peers on the development of self-concepts for Chinese late adolescents. This study can shed new light on the understanding of the development of self-concepts for late adolescents under different cultural backgrounds.

“The one who knows others is wise, and the one who knows oneself is really intelligent”—Lao-Tzu

## Introduction

To pursue proper self-knowledge is one of the most important tasks for an individual in his or her lifetime, especially at the stage of adolescence. Adolescence is regarded as a period that is rapid and greatly shifty, involving biological growth, changes in major social roles, and other factors (Sawyer et al., 2018). Despite the historical and cultural differences in the definition of adolescence, modern scholars generally define it as the period between the ages of 10 and 24 years (Sawyer et al., 2018; Crone and Fuligni, 2020). With the enhancement of individual autonomous awareness during adolescence (Steinberg and Silverberg, 1986) and the assumption of new social roles and changes in the living environment (Brown, 2004; Harter, 2015), self-exploration and identity development become the most important developmental tasks at this stage (Erikson, 1963). Therefore, scholars generally think that adolescence is a stage of lifetime during which self-knowledge changes significantly (Harter, 2015; Romund et al., 2016; Cruijsen et al., 2018).

Self-knowledge is considered to be a collection of self-representations that can be truly and accurately described (Bukowski, 2019), which not only involves how an individual usually thinks and feels, as well as the self-perception of behavior, but also refers to how an individual interprets the awareness of these patterns to others (Vazire, 2010; Xua et al., 2015). On the one hand, self-knowledge of people needs to depend on individual introspection, that is, to survey our inner world (such as feelings, goals, and memories) (Bukowski, 2019). On the other hand, self-knowledge also roots in the social interaction (Hinde et al., 2001; Bollich et al., 2011; Harter, 2015). The classical theory of symbolic interaction (Cooley, 1902; Mead, 1934) emphasizes the social construction of the self which believes that the social interaction plays an important role in the construction, maintenance, and change of the self (Kaufman and Johnson, 2004). Without the interaction with others, people would certainly not have a self-view (Swann and Bosson, 2010).

Reflected appraisals, which are the perception of an individual of how others view them, are the core of studying how the social interaction affects the self in the theory of symbolic interaction (Gecas and Burke, 1995; Srivastava, 2012). The reflected appraisals model holds that when others make judgments about us (i.e., actual appraisals of others), we will perceive appraisals of others on us (i.e., reflected appraisals), and then, we will internalize the perceived appraisals into our own view on ourselves (i.e., self-appraisals; Kinch, 1963; Stets et al., 2020). Some studies have proved the mediating role of reflected appraisals in different fields, such as the influence of parents, teachers, and classmates on the academic ability of middle-school students (Bouchey and Harter, 2005; Nurra and Pansu, 2009; Tomasetto et al., 2015); the influence of classmates on the teaching ability of normal University students (Hu et al., 2014); and the influence of parents, coaches, and teammates on the sports ability of teenagers (Amorose, 2002, 2003; Bois et al., 2005); the influence of parents or peers on criminal behavior (Brownfield and Thompson, 2005; Walters, 2016); the influence of social environment on racial identity (Khanna, 2010; Sims, 2016); and the influence of parents on the self-concept of adolescents (Silva et al., 2020). However, some studies did not underpin the mediation hypothesis of reflected appraisals (Felson, 1993; Hergovich et al., 2002).

People interact socially with many different others, but different others are of different importance to an individual. If the other is regarded as the relevant, important, valuable, expected, and a member of the group by the individual, the perception of appraisals of another person is more likely to be internalized into the self-concept (Cast et al., 1999; Sinclair et al., 2005; Srivastava, 2012; Wallace and Tice, 2012). On the one hand, at the stage of adolescence, individuals are strongly influenced by peers (Borghuis et al., 2017; Luan and Bleidorn, 2019; Crone and Fuligni, 2020); they spend significantly less time on their parents, but significantly more time on their peers (Jankowski et al., 2014); they are more sensitive to the acceptance or rejection of information by peers (Pfeifer and Peake, 2012), especially of their friends or lovers (Yue et al., 2012, 2020). On the other hand, adolescents have not yet fully formed a stable self-view (Erikson, 1963), and even in the late adolescence (i.e., 18–24 years old), their main feature is also to explore their identity (Veroude et al., 2014). Moreover, with the development of cognitive ability of an individual, the change of living environment, and other aspects during adolescence, adolescents form more and more abstract self-descriptions, and more different self-concepts (Brown, 2004; Harter, 2015); and self-representation increasingly focuses on interpersonal or social characteristics (Lu, 1990).

Under different cultural backgrounds, self-concept of people may not be affected by social others to the same degree. Individualist culture attaches more importance to the independence and uniqueness of individuals, while collectivist culture attaches more importance to interpersonal relations and interdependence (Triandis, 1995), thus forming independent self-construct and interdependent self-construct, respectively (Markus and Kitayama, 1991). Some studies have found that, compared with Americans, Chinese are better at perspective taking (Wu and Keysar, 2007). In particular, when appraising oneself in a field highly related to the evaluation of others, individuals with interdependent self-construct have more perspective taking (Li, 2006; Pfeifer et al., 2017). For people with individualistic tendency, their self-representation is more likely to be constructed in a general way, while for people with collectivist tendencies, their self-representation is more likely to be constructed in a context mode (Zhou and Cacioppo, 2010). Some scholars believe that, compared with Americans, the self-concepts of Japanese college students are more affected by the presence of others (Kanagawa et al., 2001). Some studies have also found that the perspective of others very often becomes the default position of the East Asian self (Suh, 2007). These studies meant that the appraisals of others have a greater influence on individual self-concept in the collectivist culture. Therefore, the first issue to be explored in this study is whether peers influence the self-perception of Chinese adolescents, and if so, what role does reflected appraisals play in this?

Cultural factors are not only reflected in the self-concept of people but also in the process of reflected appraisals. Researchers believe that reflected appraisals are interlocking series of processes (Wallace and Tice, 2012), which involve both how others express their appraisals on an individual and how an individual receives such feedback information, even how an individual accurately perceives views of others on himself or herself. In general, people do not express negative information directly (DePaulo and Bell, 1996). Since Chinese culture focuses more on interpersonal harmony (Ho et al., 1991; Yang, 1995; Kim et al., 2006), others will be more indirect and implicit when expressing appraisals information (Gao, 1998; Wen et al., 2009; Hu et al., 2014), which may mean that others usually express more positive views toward individuals. Given the characteristics of Chinese culture, the second issue discussed in this study is whether individuals in the context of Chinese culture can accurately perceive the views of others of them. If not, what are the characteristics of their self-concepts?

One of the salient features of Chinese culture is the worship of modesty (Cai et al., 2011); its main manifestation is “humble oneself and respect others” (Hu and Huang, 2006). That is to say, in order to maintain interpersonal harmony in interpersonal interaction, modesty requires individuals to put themselves in a relatively low position and others in a higher position and use some low-key, implicit way to show themselves (Hu and Huang, 2009), and even to some extent self-deprecating (Shi and Zhang, 2018). This means that modesty requires individuals to keep a low profile, both in self-evaluation and in inferring what others think of them, which in turn showed lower self-appraisals and reflected appraisals. Therefore, we proposed Hypothesis 1: Chinese late adolescents may underestimate the views of others on themselves.

Besides, according to the self-other knowledge asymmetry (SOKA) model (Vazire, 2010), the self and the others have similar information about individuals in some fields of high observations and low evaluativeness (such as extraversion). In the fields of low observations and evaluativeness (such as emotional stability), the self has more information. In the fields of low observations and high evaluativeness (such as openness), others have more information. Therefore, we proposed Hypothesis 2: The relationship among the actual appraisals, reflected appraisals, and self-appraisals of others varies with different traits.

Scholars generally believe that Chinese people have interdependent self-construct (Zhu et al., 2007; Pfeifer and Peake, 2012; Ma et al., 2014), while some studies point out that the degree of interdependent self-construct of Chinese people depends on different fields, with more interdependence in the social self-field and more independence in the academic field (Zhang et al., 2006; Pfeifer et al., 2017). Therefore, this study mainly focuses on the social field of Chinese self. Vazire (2010) believed that most of the personality is an interpersonal relationship in essence. This study took the Big Five personality as the content of appraisals, to explore the influence of peers on the self-concept of Chinese late adolescents. Due to the relative instability of the self in adolescence, peers become more and more important. On this basis, we proposed Hypothesis 3: Actual appraisals of others indirectly affect the self-appraisals of individuals through reflected appraisals.

## Methods

### Participants

According to the definition of adolescence (Sawyer et al., 2018; Crone and Fuligni, 2020), the ages of adolescence range from 10 to 24 years. Therefore, we recruited Chinese late adolescents aged from 18 to 24 years. In this study, 59 undergraduate students were recruited *via* the internet through convenient sampling [32 women, 19–23 years old, *M* = 21.06, standard deviation (*SD*) = 1.06]. They were given a detailed introduction and received a written consent prior to the study. They received course credits for their participation. Then, each participant was told to complete three types of questionnaires, namely, self-appraisal questionnaire, reflected appraisal questionnaire, and actual appraisals of peers questionnaire. A total of 387 questionnaires were distributed, and all of them were completed and qualified. This study was approved by the Ethics Committee of the Chongqing University of Arts and Sciences.

### Procedure

According to previous research procedures (Levesque, 1997; Hu et al., 2014), 59 participants were divided into 16 groups, each group consisting two to four members who were familiar with each other. Members in each group came to the lab together and completed the study at the same time. In the lab, everyone sat at a desk with a partition, which is to prevent each other from communicating when filling in the questionnaires. The participants were asked to perceive their personality traits based on the Big Five Inventory. They completed three types of questionnaires in order, namely, (a) self-appraisal questionnaire (i.e., how they perceived of themselves), (b) reflected appraisal questionnaire (i.e., how they perceived his or her peers in the group evaluating their own personality traits), and (c) actual appraisals of others questionnaires (i.e., evaluating personality traits of his or her peers in the group). The number of questionnaires completed by each person is ([2 × the number of people in the group] −1).

### Measures

We adopted the 44-item version of Big Five Inventory (John and Srivastava, 1999), which has been proved to be suitable for personality measurement in the context of Chinese culture (Li et al., 2015; Carciofo et al., 2016). It consists of five factors, namely, openness, conscientiousness, extroversion, agreeableness, and emotional stability. The 44 items were assessed on a 7-point Likert scale ranging from 1 (i.e., does not apply at all) to 7 (i.e., applies fully). The Cronbach's alpha coefficients for the subscales of the Big Five Inventory ranged from 0.75 to 0.82. The classical paradigm from previous studies (Nurra and Pansu, 2009; Silva et al., 2020) was adopted to measure self-appraisals, reflected appraisals, and actual appraisals of others. Example items included “Am I talkative?” (i.e., self-appraisals), “Does my peer think I'm talkative?” (i.e., reflected appraisals), and “Is my peer talkative?” (i.e., actual appraisals of peers and the names of peers of each participant were written here).

### Statistical Analysis

First, the self-appraisals, reflected appraisals, and actual appraisals of peers of each participant were matched one by one. A total of 164 groups of data were processed and analyzed by using the SPSS version 18.0 software. The descriptive statistics (i.e., mean and SD), Pearson correlations, and repeated-measures analysis of variance (ANOVA) were computed. Subsequently, the SPSS macro PROCESS Model 4 (Hayes and Preacher, 2013) was adopted for the mediation analysis, with self-appraisals as the independent variable, reflected appraisals as the mediator, and the actual appraisals of peers as the outcome variable; 5,000 bias-corrected bootstrapped resamplings were used to estimate the 95% confidence interval (CI). Mediation was deemed to be statistically significant if the CIs did not include zero.

## Results

### The Test of Common Method Variance

Identifying common methods variance with the data collected from a single source was considered as a sticky issue (Avolio et al., 1991). In the present study, we used the Harman's one-factor test (Podsakoff and Organ, 1986) to analyze the common methods variance (Livingstone et al., 1997). The basic assumption of this technique was that if a large number of method variations are present, then a single factor would be isolated during the factor analysis or a common factor explained most of the variation (Fuller et al., 2016). In this study, unrotated factor analysis was carried out to analyze 13 factors of characteristic roots above 1, and the first principal factor explained 17.49% of the variation, which was <40%. The result suggested that there was not any obvious common method bias.

### The Differences and Correlations Between the Three Types of Appraisals of Big Five Personality

Descriptive statistics (i.e., mean and SD) and correlations between all variables are summarized in Table 1. In order to compare the differences between his/her self-appraisals, reflected appraisals, and actual appraisals of peers of Big Five personality traits, we conducted a repeated measure ANOVA. In the ANOVA analysis, the appraisal condition (i.e., self-appraisals, reflected appraisals, and actual appraisals of peers) was considered as a within-subject factor. The results yielded significant main effects of appraisal condition on openness [*F*_(2, 326)_ = 5.41, *p* < 0.01, η^2^ = 0.032], conscientiousness [*F*_(2, 326)_ = 20.50, *p* < 0.001, η^2^ = 0.112], extroversion [*F*_(2, 326)_ = 31.62, *p* < 0.001, η^2^ = 0.162], and emotional stability [*F*_(2, 326)_ = 39.74, *p* < 0.001, η^2^ = 0.196] but not on agreeableness [*F*_(2, 326)_ = 1.99, *p* > 0.05]. Further, the one-way ANOVA and *post-hoc* test were conducted, and the results showed the general trend on extroversion, conscientiousness, and emotional stability that the scores of actual appraisals of peers were significantly higher than that of reflected appraisals and self-appraisals, and the scores of reflected appraisals were significantly higher than that of self-appraisals. For openness, the scores of actual appraisals of peers were significantly higher than that of reflected appraisals and self-appraisals, but there was no significant difference between reflected appraisals and self-appraisals.

**Table 1:** The descriptive statistics and correlations between three types of appraisals.

**Factors**	**SA**	**RA**	**AA**	**SA-RA**	**RA-AA**	**SA-AA**
	**M (*SD*)**	**M (*SD*)**	**M (*SD*)**			
Extroversion	4.03 (0.93)	4.31 (0.99)	4.55 (0.91)	0.77^**^	0.56^**^	0.48^**^
Agreeableness	4.82 (0.64)	4.75 (0.58)	4.87 (0.69)	0.72^**^	0.25^**^	0.10
Conscientiousness	4.13 (0.78)	4.28 (0.76)	4.55 (0.68)	0.68^**^	0.21^**^	0.10
Emotional stability	3.84 (0.81)	4.13 (0.75)	4.43 (0.67)	0.69^**^	0.23^**^	0.08
Openness	4.35 (0.65)	4.25 (0.64)	4.45 (0.75)	0.73^**^	0.20^*^	0.19^**^

*SA, self-appraisals; RA, reflected appraisals; AA, actual appraisals of peers; M, mean; SD, standard deviation; SA–RA means the relationship between SA and RA*;

* *p < 0.05 and*

** *p < 0.01*.

As indicated in Table 1, the Pearson correlation analysis showed there were significant high correlations between self-appraisals and reflected appraisals on five factors (*r* = 0.68–0.77). Furthermore, for extroversion, there was a significant medium correlation between reflected appraisals and actual appraisals of peers (*r* = 0.56), whereas reflected appraisals and actual appraisals of peers showed significantly low relationships on the other four factors (*r* = 0.20–0.25). There was a significant medium correlation between self-appraisals and actual appraisals of peers (*r* = 0.48) on extroversion and a low correlation on openness (*r* = 0.19), but no significant correlation on the other three factors.

### The Mediation Analyses

**Figure 1:**
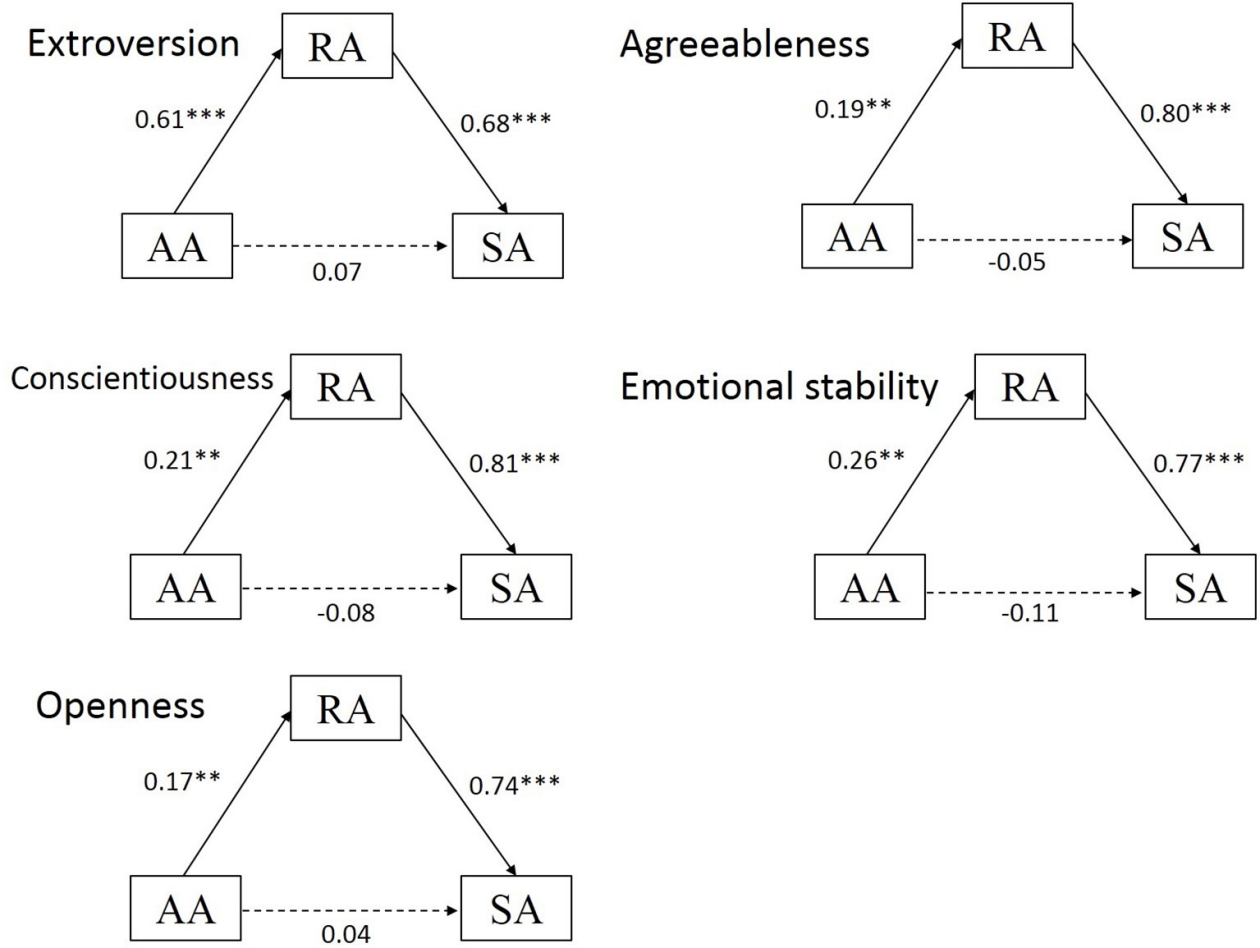
Mediation models of the effect of actual appraisals of peers and self-appraisals via reflected appraisals. SA, self-appraisals; RA, reflected appraisals; AA, actual appraisals of peers; M, mean; **p < 0.01 and ***p < 0.001.

We adopted the model 4 in the SPSS macro PROCESS (Hayes and Preacher, 2013) to test the mediating effect of reflected appraisals on the influence of actual appraisals of peers on self-appraisals. The results showed that reflected appraisals were significantly related to self-appraisals (extroversion, β = 0.73, *p* < 0.001; agreeableness, β = 0.74, *p* < 0.001; conscientiousness, β = 0.69, *p* < 0.001; emotional stability, β = 0.71, *p* < 0.001; openness, β = 0.72, *p* < 0.001). The bias-corrected percentile bootstrap analysis further revealed that the mediation effects of reflected appraisals on the relationship between actual appraisals of peers and self-appraisals on the Five Personality traits were all significant (see Table 2 and Figure 1). The indirect effects of the mediating variable were as follows: extroversion [*ab* (means indirect effect value) = 0.42, standard error (*SE*) = 0.06, 95% CI = 0.31–0.54], agreeableness (*ab* = 0.17, *SE* = 0.05, 95% CI = 0.07–0.29), conscientiousness (*ab* = 0.15, *SE* = 0.06, 95% CI = 0.05–0.26), emotional stability (*ab* = 0.20, *SE* = 0.07, 95% CI = 0.06–0.34), and openness (*ab* = 0.12, *SE* = 0.05, 95% CI = 0.04–0.23).

**Table 2:** Direct effect, indirect effect, and total effect among the variables.

		**Effect**	**Boot**	**Boot**	**Boot**
			**SE**	**LL CI**	**UL CI**
Extroversion	Indirect effect	0.42	0.06	0.31	0.54
	Direct effect	0.07	0.06	−0.05	0.20
	Total effect	0.49	0.07	0.35	0.64
Agreeableness	Indirect effect	0.17	0.05	0.07	0.29
	Direct effect	−0.08	0.05	−0.19	0.02
	Total effect	0.09	0.07	−0.05	0.23
Conscientiousness	Indirect effect	0.15	0.06	0.05	0.26
	Direct effect	−0.05	0.07	−0.18	0.08
	Total effect	0.10	0.09	−0.07	0.27
Emotional stability	Indirect effect	0.20	0.07	0.06	0.34
	Direct effect	−0.11	0.07	−0.24	0.03
	Total effect	0.09	0.09	−0.10	0.27
Openness	Indirect effect	0.12	0.05	0.04	0.23
	Direct effect	0.04	0.04	−0.04	0.13
	Total effect	0.17	0.07	0.04	0.29

*Bootstrap sample size = 5,000; SE, standard error; LL, low limit; UL, upper limit; CI, confidence interval*.

## Discussion

This study mainly discussed the influence of peers on self-perception during late adolescence under the background of collectivistic culture, and the results showed that reflected appraisals played a mediating role between actual appraisals of peers and self-perception, that is, actual appraisals of peers indirectly influences self-perception of adolescents through reflected appraisals. This study also found that there was a certain degree of correlation between reflected appraisals of an individual and actual appraisals of peers under the background of collectivistic culture, but the score of reflected appraisals was lower than actual appraisals of peers, which indicated that Chinese people had a certain degree of accuracy of views of others on themselves that was usually underestimated by themselves.

The study has found that, on the dimensions of extroversion, conscientiousness, and emotional stability, self-appraisals were lower than reflected appraisals and actual appraisals of peers; meanwhile, reflected appraisals were lower than actual appraisals of peers. Furthermore, Chinese people had the least positive views on themselves and views of others on themselves, which were different from the research on Western individuals. Research on the accuracy of reflected appraisals in Western culture finds that in terms of personality traits, scores of reflected appraisals are higher than actual evaluation of others, indicating that Westerners overestimate the views of others on themselves (Carlson and Kenny, 2012). We speculated that this result reflects cultural characteristics of self. From the perspective of social interaction, Chinese culture emphasized on interpersonal harmony and coexistence (Ho et al., 1991; Yang, 1995; Kim et al., 2006), and Chinese people paid attention to “face” in the process of social interaction (Wen et al., 2009), which determined the implicit and indirect of expression in the process of interpersonal communication (Gao, 1998; Hu et al., 2014). Therefore, in the process of interpersonal interaction, in order to maintain the “face” of each other (Wen et al., 2009), Chinese people usually do not directly express the feedback about the actual situation of others but give more positive appraisals feedback on them. In contrast, since Chinese culture advocates modesty, which requires showing “humble oneself and respect others” in interpersonal interaction, Chinese people do not show their uniqueness too much in interpersonal interaction but degrade themselves to a certain extent (Shi and Zhang, 2018), showing low self-appraisals. Given the rules that preserve the “face” of others in interpersonal interactions and the cultural norms that require modesty about oneself, reflected appraisals of Chinese people are inaccurate and may underestimate perceptions of others of themselves. However, this study found that on the agreeableness dimension, there was no significant difference in the three kinds of appraisals scores. This result could suggest the self-enhancement motivation (not a self-modest way) of Chinese people on the agreeableness dimension. Some scholars pointed out that when it came to the most meaningful and important part of the self, East Asians would show self-enhancement motivation (Wang, 2005). As Chinese culture focused on interpersonal relationships, Chinese regarded the self as social roles and relationships of a person (Zhu et al., 2007), suggesting that interpersonal relationship for Chinese self was one of the most important and meaningful contents. Therefore, they showed higher scores of self-appraisals on the agreeableness dimension, and there was no significant difference between three types of appraisals.

This study also found that there was a high correlation between Chinese self-appraisals and reflected appraisals and a significant low correlation between reflected appraisals and actual appraisals of peers, which was in accord with the existing results (Silva et al., 2020), indicating that Chinese are able to know their views of peers on them to a certain extent. This study also discovered that the relationships between the three types of appraisals varied with the different traits. Specifically, the relationships between the three types of appraisals were related to trait characteristics. On the dimension of extraversion, actual appraisals of peers had medium to high correlations with reflected appraisals and self-appraisals, whereas, actual appraisals of peers had a significant low correlation with self-appraisals and reflected appraisals on openness. For agreeableness, conscientiousness, and emotional stability, there was a significant low correlation between reflected appraisals and actual appraisals of peers and no significant correlation between self-appraisals and actual appraisals of peers. These results could be related to the self–other knowledge asymmetry model (Vazire, 2010). According to the SOKA model, personality traits involved both observability and evaluativeness. Extroversion was a field with a high observability but low evaluativeness; the judgments of self and others were based on similar information (i.e., observation of external behaviors of an individual); therefore, there are high correlations between the three types of appraisals. Openness is a field with a high evaluativeness but low observability, which indicates that others have more information, and self-information of openness is more from outside others, so the self could partially detect actual appraisals of peers. Accordingly, there were significant correlations between self-appraisals, actual appraisals of peers, and reflected appraisals. In the SOKA model, emotional stability is a field with a low observability and evaluativeness, which indicates that individuals have more information about themselves on this dimension, while others have less relevant information, thus, self-appraisals do not show a significant correlation with actual appraisals of peers. However, under the background of collectivistic culture, agreeableness and conscientiousness have a high social desirability (Schlicht et al., 2009). We tended to show bias when evaluating personality traits with a high social desirability (Chen et al., 2013), which may lead to the non-significant correlation between self-appraisals and appraisals of others.

In this study, it was found that actual appraisals of peers had an impact on the self-perception of Chinese in late adolescence, but this impact was indirectly influenced by reflected appraisals, which verified the effectiveness of reflected appraisals model under collectivist culture. On the one hand, Chinese culture attached great importance to interpersonal relationship (Zhu and Han, 2008), and Chinese people had interdependent selves. On the other hand, with the growth of the individuals, peers have become increasingly important (Borghuis et al., 2017; Luan and Bleidorn, 2019), so individuals are sensitive to the feedback of peers (Pfeifer and Peake, 2012). Moreover, the participants in this study are in late adolescence, and their self-concepts are not stable (Veroude et al., 2014). Taken together, these factors accorded for the impact of actual appraisals of peers on his/her self-perception. It is worth noting that actual appraisals of peers did not directly affect self-appraisals under the background of Chinese culture but indirectly affected self-appraisals through reflected appraisals. Previous studies have found that Chinese were better at perspective-taking (Wu and Keysar, 2007), especially when it came to the relative fields of evaluations of others (Pfeifer et al., 2017). The appraisals content of this study was the Big Five personality traits, and Vazire and Carlson (2010) believed that most of the personality was an interpersonal relationship in essence, which meant that Chinese had stronger perspective taking in the judgment of the Big Five personalities. Generally, perspective taking was the basis of reflected appraisals. Previous studies found that adolescents usually pay more attention to the views of others (Pfeifer et al., 2009; Harter, 2015); especially in East Asia, views of others were the default position of the self (Suh, 2007). Therefore, regardless of the accuracy of reflected appraisals of individuals, the self-knowledge of Chinese was based more on reflected appraisals in the process of interpersonal interaction. Accordingly, reflected appraisals played an intermediary role between actual appraisals of peers and self-appraisals.

This study explores the relationship between self-appraisals, reflected appraisals, and actual appraisals of others on the Big Five personality traits of Chinese people, extending this research field to collectivist culture, and finds the cultural characteristics of reflected appraisals. On the one hand, this study further verifies the rationality of the reflected appraisals model under the collectivism culture. Specifically, actual appraisals of peers indirectly affect the self-appraisals of Chinese teenagers through reflected appraisals. On the other hand, the study also found the uniqueness of reflected appraisals in the context of Chinese culture. Due to the cultural requirements of “face” and “modesty” in interpersonal interaction, Chinese people are more low-key in reflected appraisals compared with Westerners and tend to underestimate the opinions of their peers of them. This study is helpful to understand the formation process of personality of people under collectivism culture. However, this study also has some limitations. First, in terms of the appraisals content, this study only involved the Big Five personality traits. Some studies believed that the self-construct of Chinese subjects is related to specific fields, and there was an independent self-construct in the academic field (Pfeifer et al., 2017). Therefore, whether the results of this study are applicable to the academic field still needs to be further discussed. Second, the participants in the present study were in late adolescence, and developmental psychologists generally believed that peers had a greater influence on the self-concept of individuals in early adolescence (Jankowski et al., 2014). So, further research is needed to determine whether the results can be inferred to other groups of subjects. Third, this study only selected peers groups and neglected other important people. Research on the cognitive structure of the self-found that the self of Chinese included mother (Zhang et al., 2006). Therefore, parents were also important others for Chinese. Then, under the collectivistic culture, how do reflected appraisals from parents affect the self-concept of individuals? More research is needed in the future.

In conclusion, although this study has some limitations, there are still some significant results. We found that, unlike Westerners overestimate the views of others on themselves, Chinese people often underestimate the views of others on themselves. The relationship between self-appraisals, reflected appraisals, and actual appraisals of others varies with different traits. Actual appraisals of others indirectly affect the self-appraisals of Chinese late adolescents through reflected appraisals. This study has been helpful for us to understand the characteristics and functions of reflected appraisals from a cultural perspective.

## Data Availability Statement

The original contributions presented in the study are included in the article/supplementary material, further inquiries can be directed to the corresponding author/s.

## Ethics Statement

The studies involving human participants were reviewed and approved by Ethics Committee of the Chongqing University of Arts and Sciences. The patients/participants provided their written informed consent to participate in this study.

## Author Contributions

CY and ZY designed the experiments. ZY and YL carried out the survey. CY, QX, and WP analyzed the data. CY, YL, and WP drafted the initial manuscript and revised the manuscript. All authors approved the final manuscript as submitted and agreed to be accountable for all aspects of the work.

## Conflict of Interest

The authors declare that the research was conducted in the absence of any commercial or financial relationships that could be construed as a potential conflict of interest.

## Publisher's Note

All claims expressed in this article are solely those of the authors and do not necessarily represent those of their affiliated organizations, or those of the publisher, the editors and the reviewers. Any product that may be evaluated in this article, or claim that may be made by its manufacturer, is not guaranteed or endorsed by the publisher.
